# An artificial intelligence algorithm to select most viable embryos considering current process in IVF labs

**DOI:** 10.3389/frai.2024.1375474

**Published:** 2024-05-30

**Authors:** Mahdi-Reza Borna, Mohammad Mehdi Sepehri, Behnam Maleki

**Affiliations:** ^1^Department of IT Engineering, Faculty of Industrial and Systems Engineering, Tarbiat Modares University, Tehran, Iran; ^2^Infertility Center, Department of Obstetrics and Gynecology, Mazandaran University of Medical Sciences, Sari, Iran; ^3^Research and Clinical Center for Infertility, Yazd Reproductive Sciences Institute, Shahid Sadoughi University of Medical Sciences, Yazd, Iran

**Keywords:** artificial intelligence, deep learning, embryo selection, *in-vitro* fertilization, medical images

## Abstract

**Background:**

The most common Assisted Reproductive Technology is *In-Vitro* Fertilization (IVF). During IVF, embryologists commonly perform a morphological assessment to evaluate embryo quality and choose the best embryo for transferring to the uterus. However, embryo selection through morphological assessment is subjective, so various embryologists obtain different conclusions. Furthermore, humans can consider only a limited number of visual parameters resulting in a poor IVF success rate. Artificial intelligence (AI) for embryo selection is objective and can include many parameters, leading to better IVF outcomes.

**Objectives:**

This study sought to use AI to (1) predict pregnancy results based on embryo images, (2) assess using more than one image of the embryo in the prediction of pregnancy but based on the current process in IVF labs, and (3) compare results of AI-Based methods and embryologist experts in predicting pregnancy.

**Methods:**

A data set including 252 Time-lapse Videos of embryos related to IVF performed between 2017 and 2020 was collected. Frames related to 19 ± 1, 43 ± 1, and 67 ± 1 h post-insemination were extracted. Well-Known CNN architectures with transfer learning have been applied to these images. The results have been compared with an algorithm that only uses the final image of embryos. Furthermore, the results have been compared with five experienced embryologists.

**Results:**

To predict the pregnancy outcome, we applied five well-known CNN architectures (AlexNet, ResNet18, ResNet34, Inception V3, and DenseNet121). DeepEmbryo, using three images, predicts pregnancy better than the algorithm that only uses one final image. It also can predict pregnancy better than all embryologists. Different well-known architectures can successfully predict pregnancy chances with up to 75.0% accuracy using Transfer Learning.

**Conclusion:**

We have developed DeepEmbryo, an AI-based tool that uses three static images to predict pregnancy. Additionally, DeepEmbryo uses images that can be obtained in the current IVF process in almost all IVF labs. AI-based tools have great potential for predicting pregnancy and can be used as a proper tool in the future.

## 1 Introduction

Infertility affects approximately 186 million people worldwide, affecting an estimated 8–12 percent of child-bearing couples (Inhorn and Patrizio, 2015). In recent decades, many couples have turned to *in-vitro* fertilization (IVF) to help them conceive. IVF includes controlled ovarian stimulation, egg retrieval, sperm preparation, fertilization, embryo culture in a laboratory for 1–6 days, and embryo transfer to the uterus of patients.

Despite significant developments in IVF technology over the last decade, the success rate remains below expectations (Dyer et al., 2016). Only 10–30% of transferred embryos result in a live delivery, and many patients require multiple cycles to become pregnant (Wang and Sauer, 2006; Doostabadi et al., 2022; Harrison et al., 2022).

Although many factors influence the success of IVF cycles, including medical diagnosis, maternal age, the quality of embryo and gamete, and endometrium receptivity, the embryo selection process is one of the essential key factors to ensuring a successful pregnancy and ensuring the patient’s shortest time to pregnancy (Minasi et al., 2016).

Increasing the number of embryos transferred per cycle can increase the chances of pregnancy. However, the chances of having multiple pregnancies as a most significant risk of Assisted Reproductive Technology (ART) also increase. Selection of the best embryo and Single Embryo Transfer (SET) has been proven to decrease multiple pregnancy. The most prevalent approach for best embryo selection is the morphological evaluation of embryos using an optical light microscope by experienced embryologists (Machtinger and Racowsky, 2013). The primary disadvantages of this procedure are its subjective nature and intra- and inter-operator variability among embryologists of various skill levels (Baxter Bendus et al., 2006; Storr et al., 2017).

Furthermore, despite extensive research and many suggested embryo grading systems, no agreement on the best reliable methodology for predicting pregnancy has been reached. Some technology has been introduced to help better assessments of embryos, such as the time-lapse imaging (TLI) system that enables continuous observation of embryo growth without disrupting the embryo’s micro-environment. However, there might be a lot of variability in the decisions made by embryologists to select the best embryo based on time-lapse images (Kirkegaard et al., 2015). Furthermore, Time-lapse microscopy is unavailable in every IVF laboratory and for each patient (Dolinko et al., 2017).

Because of image-based diagnosis and decision-making difficulties, computer-based prediction models based on artificial intelligence (AI) for analyzing human embryo images have recently received interest (Rad et al., 2020). Quantitative assessment of embryo parameters using images can increase success, eliminate mistakes, and lead to quicker, low-priced, and more accessible results, leading to a more accurate prediction of embryo development and implantation potential. The AI algorithm might learn how embryos develop over time and utilize that knowledge to choose the best embryos improving objectivity throughout the embryo selection process. AI tools are quick and have a consistent standard in every laboratory (Bormann et al., 2020). Moreover, AI systems may be able to discover previously unknown connections between different characteristics of embryos. AI systems also offer significant economic benefits to healthcare systems (Borna and Sepehri, 2024).

Deep learning techniques, namely convolutional neural networks (CNNs), have recently been employed to solve various medical imaging challenges. In computer vision, CNNs have become the most popular and successful type of image analysis models. Its application in medical images includes and is not limited to polyp detection and segmentation (Ji et al., 2021), skin cancer detection (Mazoure et al., 2022), and segmentation and detection in Covid-19 X-ray images (Ahmed et al., 2021; Gourdeau et al., 2022). Similarly, there has been considerable interest in using machine learning-based algorithms to analyze embryos. Researchers in this field have focused most of their attention on using various machine learning tools to identify the best-quality embryo based on their implantation potential. Handcrafted features (human input) are required for classical machine learning techniques to work efficiently. Various classical machine learning methods are available for embryo assessment, including logistic regression models, support vector machines (SVM), Bayesian classifiers, and random forests and their combinations (Barash et al., 2018; Matsubayashi et al., 2018; Miyagi et al., 2019).

The new deep learning algorithms do not need handcrafted features and learn features at the pixel level. Deep learning algorithms have been applied to two kinds of data available in IVF labs for embryo selection. Some studies used deep learning algorithms on a single final image of embryos (Khosravi et al., 2019; Bormann et al., 2020; Chavez-Badiola et al., 2020; Huang et al., 2022). Other studies applied deep learning algorithms to time-lapse images of embryos (Lee et al., 2021; Liao et al., 2021; Sawada et al., 2021; Berman et al., 2023; Sharma et al., 2024). While deep learning approaches are more accurate on TLIs than just single images, TLI facilities are unavailable in most IVF laboratories. Furthermore, there is a notable gap in methodologies that utilize a series of images taken at different stages of embryo development which would align with the capabilities of most existing IVF labs, rather than relying solely on single-image analyses or comprehensive time-lapse systems.

This study proposes, DeepEmbryo, a non-invasive AI-based assessment algorithm to predict clinical pregnancy outcomes using three static images captured by optical light microscopy at different times post-insemination. DeepEmbryo was trained to automatically segment and use transfer learning to assess the pregnancy result of human embryos to aid in embryo selection during IVF. Using transfer learning helps us to overcome issues related to the limited data. DeepEmbryo works only based on three images that can be obtained from equipment already accessible in most IVF laboratories despite TLI. Its results are also more accurate than using just a single embryo image.

The main contribution of this paper is to provide a tool to select the best embryos without the need to change the current setting or process in IVF labs and with a better result than just using the single final image of embryos. Furthermore, we compare the performance of our models with embryologists experts in order to validate models for using in real world cases.

## 2 Methods and materials

### 2.1 Data

Data were retrospectively collected from infertile couples who had been previously diagnosed with infertility at the Research and Clinical Center for Infertility, Yazd Reproductive Sciences Institute, Shahid Sadoughi University of Medical Sciences, Yazd, Iran, from July 2017 to February 2020.

GnRH agonists/antagonists procedures were employed to induce ovarian hyperstimulation, followed by IVF/ICSI and fresh or frozen–thawed embryo transfer. A quantitative beta hCG test was done 15 days after embryo transfer. A beta hCG level of 50 mIU/mL was considered positive.

**Figure 1:**
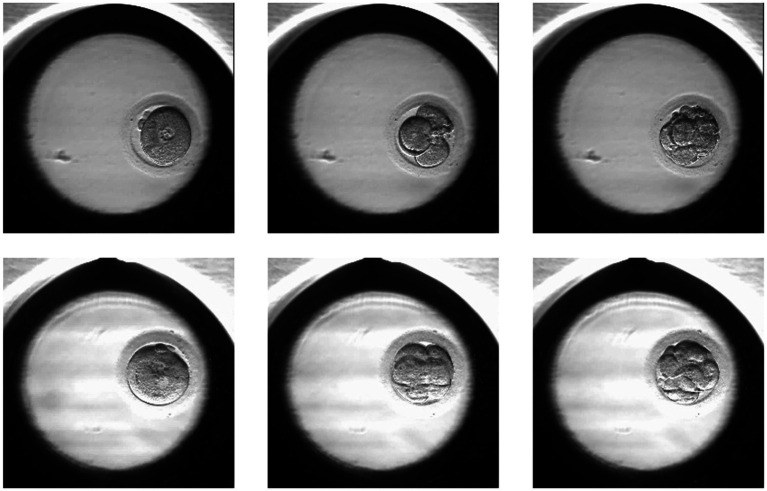
Samples of the positive (lower row) and negative (upper row) embryos in different hpi: 19 ± 1, 44 ± 1, 68 ± 1.

The images were taken at 10-min intervals with a single red LED (635 nm) using the EmbryoScope® time-lapse imaging equipment (Vitrolife, Sweden). The monitoring ended when the embryo was transferred or vitrified. Using the imaging system software, images of each patient were exported as videos. Individual embryo videos were split into frames to obtain pictures from all time points. The images were extracted at a resolution of 256 × 256 pixels. From all frame series, we extracted images related to 19 ± 1, 44 ± 1, and 68 ± 1 h post insemination (hpi). The images were then categorized into positive or negative samples (according to the pregnancy outcome). Figure 1 shows two sets of embryo images at different hpi from different classes.

The images are from time-lapse frames and were divided at the embryo level into two groups: training and testing. The training group received two-third of the images, while the test group received the remaining one-third. The training and test sets did not overlap. In other words if one of the embryos related to the patient who has two embryos is in the training data, the other embryo is definitely in the training data and not in the test data.

Image augmentation is a well-known approach for regularizing the network in supervised learning. Rotation, horizontal flip, and vertical flip are examples of general augmentations. We need a large number of training samples to reach the best results, so data augmentation is used to expand the number of original input data by creating more training data samples. A variety of randomized processes are used in augmentation, including:

- Rotating by an angle [0, 360]
- Zooming in or out [0.8, 1.2]
- Shear transform [0.8, 1.2]
- Adding light Gaussian [*m* = 0, *v* = 0. 003] or Speckle noises [*m* = 0, *v* = 0. 006]

We created 30 samples from each single input sample using various augmentation techniques such as rotation, zooming, shearing, and translation for data augmentation. As a result, the total number of training samples was increased by a factor of 30. As a result, there are 5,040 increased training samples in total (Table 1).

**Table 1:** Summary of data used in this study.

Section	Negative	Positive	Total
Train	120	48	168
Augmented train	3,600	1,440	5,040
Test	60	24	84
Total	180	72	252

### 2.2 Transfer learning

CNN networks typically require significant amounts of input images to learn features effectively and discriminate between different classes of data, depending on the difficulty of the problem at hand. One way to deal with the limited data sizes is Transfer Learning (TL).

Transfer Learning has had a lot of success in computer-aided medical image processing while being a relatively new technique. It has been used both for the classification and segmentation of medical images.

A common transfer learning technique for improving a pre-trained model to target a new task is Fine-tuning. A low learning rate is usually used to avoid overfitting, and some model parameters may need to be frozen.

Transfer learning is the key to working with small datasets, such as medical images, that are impractical to acquire in large amounts. Deep learning models require a lot of data, computing power, and time to train from scratch. To tackle these issues, pre-trained models and only fine adjustments are used.

In DeepEmbryo, since our dataset contains a limited number of images, we used transfer learning to fine-tune last onelayer of five popular pre-trained [with ImageNet weights (Deng et al., 2009)] deep neural networks. Freezing many parameters helps DeepEmbryo use the pre-trained models as a feature extractor. The pre-trained models used in DeepEmbryo were AlexNet (Krizhevsky et al., 2012), ResNet18 (He et al., 2016), ResNet36 (He et al., 2016), Inception v3 (Szegedy et al., 2016), and DenseNet-121 (Huang et al., 2017).

### 2.3 DeepEmbryo algorithm

**Figure 2:**
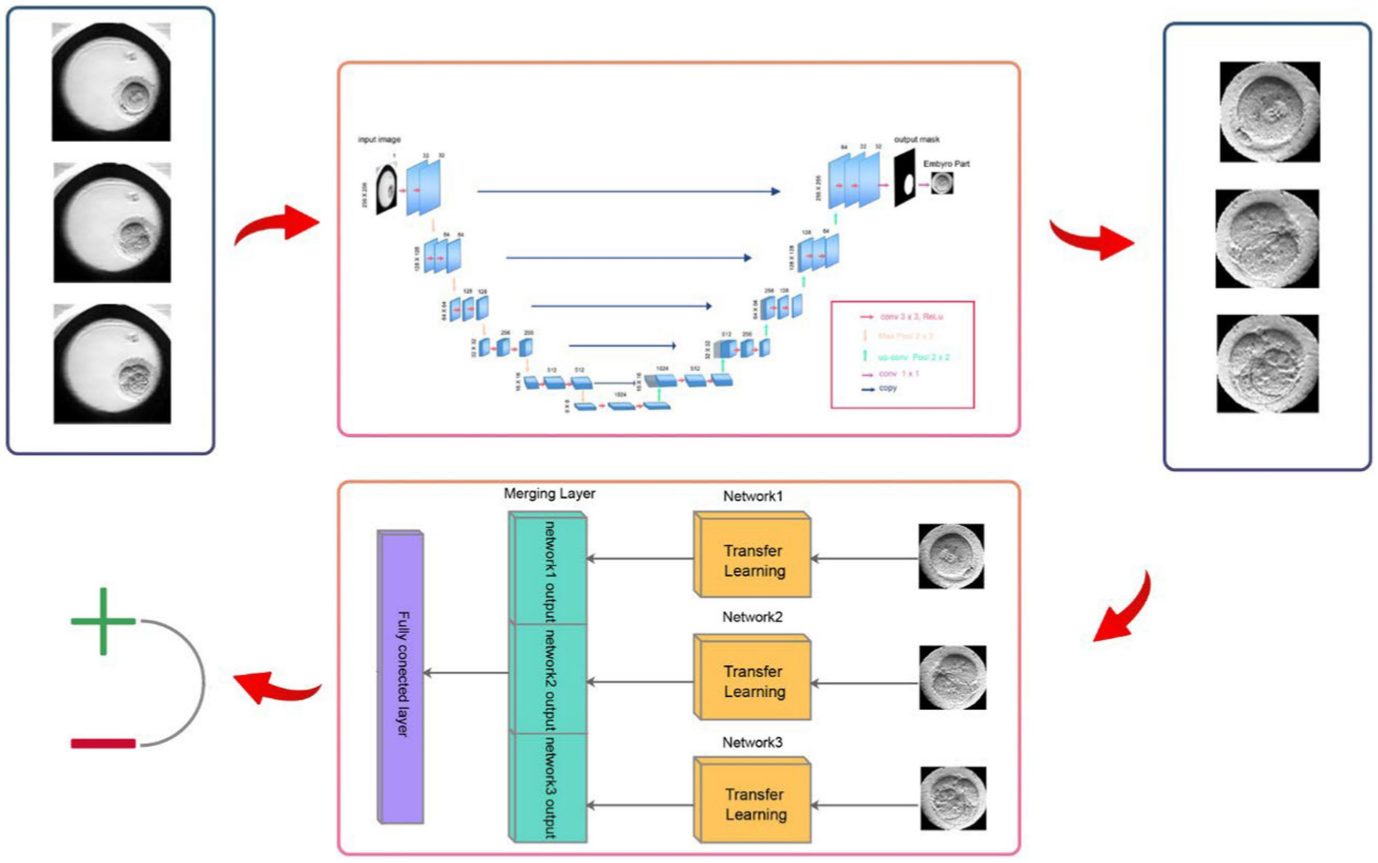
DeepEmbryo has two steps. The first step segments three input images and determines the border of the embryo parts from other parts of the images. The second step of the algorithm uses transfer learning and segmented embryos to classify input embryos.

DeepEmbryo has two Steps. The first step is the Image Segmentation Step of the algorithm. The second step uses the output of the first step and Transfer Learning to classify input embryos. Figure 2 shows an overview of the DeepEmbryo algorithm. For preparing this dataset one thousand of embryo images were annotated by an embryologist and static augmentation methods were also used. The pictures were from different frames.

### 2.4 Image segmentation step

The embryo’s segmentation can greatly help automated embryo image analysis (Rad et al., 2020). The segmentation can be thought of as a classification problem, with each pixel in the image being classified as either embryo or background. The microscope photos are the input of this phase, and embryo parts of the images are the output. After that, we used the embryo part as input for the classifier.

In our study, we utilized the U-Net architecture, specifically adapted for biomedical image segmentation, to accurately delineate the boundaries of embryos in time-lapse images (Ronneberger et al., 2015). U-Net is particularly effective for such tasks due to its architecture designed to efficiently utilize a limited amount of training data and achieve precise segmentations (Rad et al., 2020). Our implementation features a depth of five layers, which allows for an extensive feature extraction and improved image details capture, crucial for accurate segmentation.

The network architecture comprises a contracting path to capture context, characterized by a sequence of two 3 × 3 convolutions followed by a rectified linear unit (ReLU) and a 2 × 2 max pooling operation for downsampling. At each downsampling step, the number of feature channels is doubled, enhancing the network’s ability to learn complex features at different scales.

The expansive path, crucial for precise localization, includes upsampling of the feature map followed by a 2 × 2 convolution, known as “up-convolution,” which halves the number of feature channels. This is followed by concatenation with the correspondingly cropped feature map from the contracting path, and two 3 × 3 convolutions, each followed by a ReLU. This setup ensures detailed feature integration across different levels of the network.

**Figure 3:**
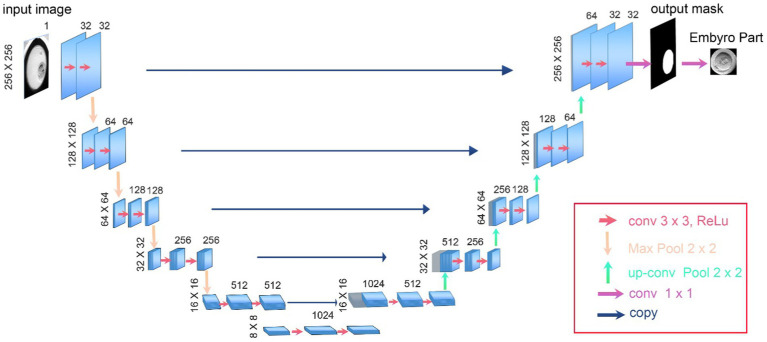
The architecture of U-Net used as the first step of DeepEmbryo for segmentation of embryo images.

The final layer of our U-Net employs a 1 × 1 convolution that provide a mask to separate background from embryo pixels. As there is exactly one embryo in each image, we used provided masks to produce segmented outputs and crop input microscopy images. After this step we will resize cropped images to fit the input of the classification algorithms. The numbers presented in Figure 3 accurately reflect the real values and parameters utilized in our U-Net model, ensuring transparency and reproducibility of our results.

### 2.5 Classification step

**Figure 4:**
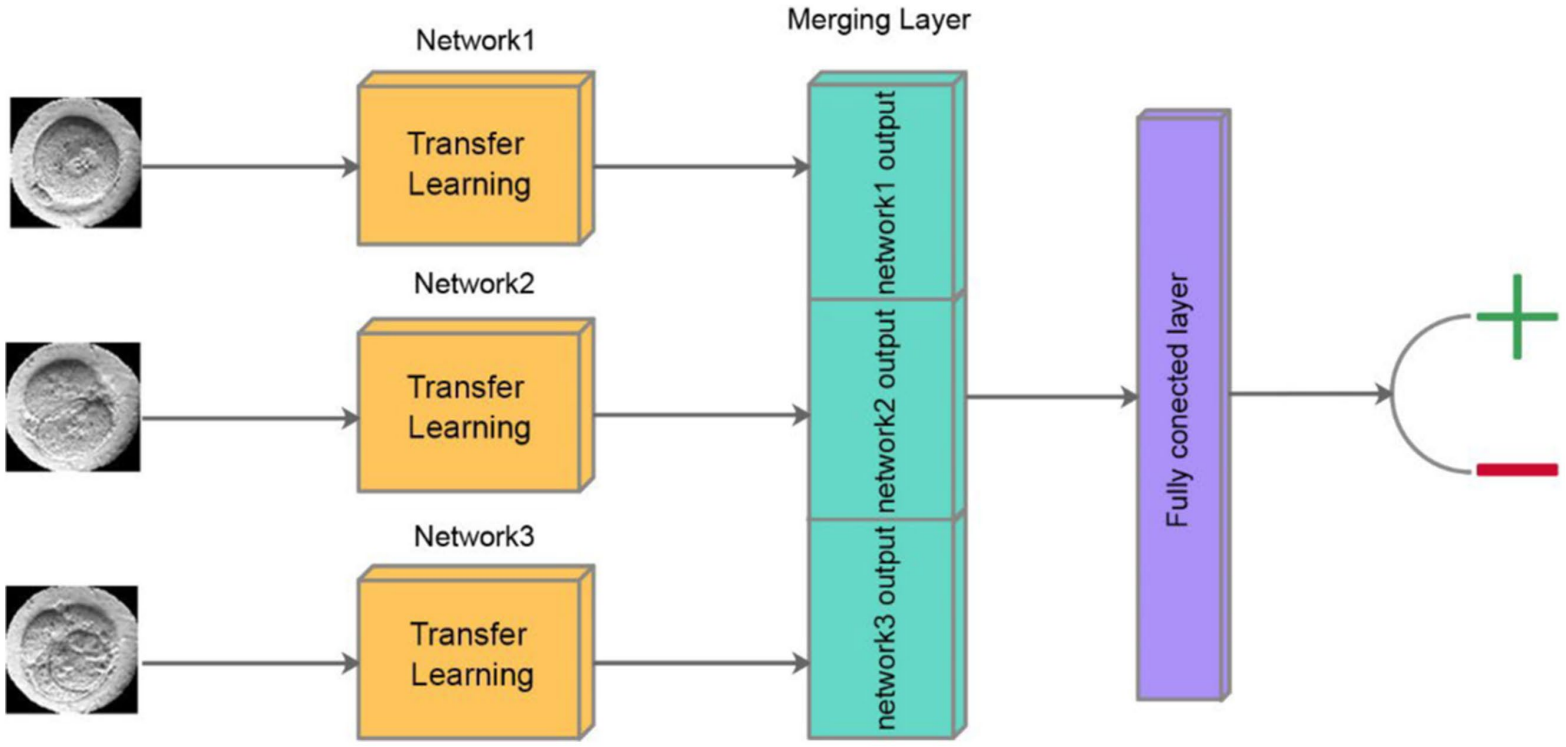
Schematic of classification step of DeepEmbryo, which uses three images and a combination of results of three pre-trained CNN to predict the outcome of pregnancy.

DeepEmbryo uses three images to predict pregnancy results. As in Figure 4, it uses a well-known CNN architecture with pre-trained weights to extract features of each image. After that, one integration layer and one fully connected layer help the algorithm learn from extracted features.

In our approach, each of the three time-lapse images undergoes separate processing through a Convolutional Neural Network (CNN). At the end of each CNN, the output layer produces a vector of size 10, encapsulating the distilled features critical for the subsequent analysis. These three vectors, each representing a different developmental stage of the embryo, are then concatenated into a single vector. This combined vector is subsequently passed through a fully connected layer, which integrates the information from all three stages to produce a final output. The output of the fully connected layer is a scalar value between 0 and 1, which represents the probability of a successful pregnancy outcome. This method ensures that the model leverages temporal insights from multiple stages of embryo development, enhancing the predictive accuracy of the system.

### 2.6 SI-DeepEmbryo

**Figure 5:**
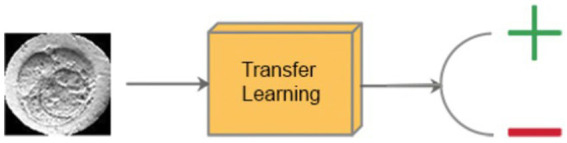
Classification step of SI-DeepEmbryo, which uses transfer learning and a single image to predict the outcome of pregnancy.

To compare with DeepEmbryo, we designed a specific model of DeepEmbryo named SI-DeepEmbryo (Single Image–DeepEmbryo), which like DeepEmbryo, has two steps; however, it uses only one final microscopic image of 68 h timpepoint embryos. Figure 5 shows the classification step of SI-DeepEmbryo.

### 2.7 Ethic

The study was approved by the Research Ethics Committees of Tarbiat Modares University (IR.MODARES.REC.1401.107). Written informed consent was obtained from all the patients for primary data collection as well as secondary analysis before starting treatment. The study followed the guidelines and protocols described in the routine practice of the IVF unit. No further interventions were used during treatment. During the data analysis, none of the authors had access to the patients information.

## 3 Results

### 3.1 Implementation details

To achieve optimal performance of the DeepEmbryo algorithm, careful consideration was given to the selection of hyperparameters. These parameters play a crucial role in the training process and the overall accuracy of the model. Below, we provide a justification for the choice of each key hyperparameter utilized in our study:

#### 3.1.1 Learning rate (0.001)

The learning rate was set at 0.001 to ensure a balance between training speed and the risk of overshooting the global minimum in the loss landscape. This value allows for gradual and stable convergence during training, minimizing the potential for erratic updates that could derail the learning process. A learning rate scheduler was also implemented to reduce the learning rate by a factor of 0.1 every 30 epochs, further aiding in fine-tuning the model’s accuracy by allowing for finer adjustments as the model approaches optimal performance.

#### 3.1.2 Batch size (32)

A batch size of 32 was chosen based on experimental trials that balanced computational efficiency with model stability. This size is large enough to ensure meaningful gradient updates and effective generalization, yet small enough to prevent excessive memory consumption and allow for more iterations per epoch, facilitating a more nuanced model training process.

#### 3.1.3 Epochs (100)

The model was trained for 100 epochs, a decision based on preliminary experiments indicating that this number of epochs was sufficient for the model to converge to a stable solution without overfitting. The chosen number of epochs ensures that the model has ample opportunity to learn from the training data across multiple passes, while the early stopping criterion (monitored on validation loss) provides a safeguard against overfitting by terminating training if the validation performance does not improve for a consecutive number of epochs.

These hyperparameters were selected through a combination of empirical testing and best practices in the field of deep learning. The configurations were iteratively refined to strike an optimal balance between model performance and computational efficiency, as evidenced by the improved accuracy and robustness of DeepEmbryo in predicting pregnancy outcomes from IVF embryo images.

### 3.2 Evaluation metrics

*Precision*, *Recall*, *Accuracy*, and *F-Score* are the four measures we use to measure DeepEmbryo’s quality. *Precision* shows how precise the model is in the detection of positive samples.


(1) $$\mathrm{Percision}=\frac{TP}{TP+FP}$$

*Recall* indicates how many of the positive instances are identified by the algorithm.


(2) $$\mathrm{Recall}=\frac{TP}{TP+FN}$$

The Accuracy of the model refers to how well it performs across all classes of data.


(3) $$\mathrm{Accuracy}=\frac{TP+TN}{TP+TN+FP+FN}$$

In all the above equations, the TP indicates positive instances correctly identified as positive, and TN measures the number of negative instances correctly identified as negative. FP is the number of negative instances incorrectly identified as positive, and FN signifies the number of positive instances that are falsely missed.

In addition to Precision, Recall, and Accuracy, the F-Score (also known as the F1-Score) is a crucial metric used to evaluate the performance of our model. The F-Score provides a harmonic mean of precision and recall, offering a balance between the two by considering both the false positives and false negatives. It is particularly useful in scenarios where an even balance between precision and recall is desired.

The F-Score is calculated using the following equation:


(4) $$F1=2\times \frac{\mathrm{Precision}\times \mathrm{Recall}}{\mathrm{Precision}+\mathrm{Recall}}$$

The F1 Score thus ranges from 0 to 1, where a higher value indicates better model performance with an ideal balance between precision and recall. This metric is particularly important in our study for evaluating the effectiveness of DeepEmbryo in accurately predicting pregnancy outcomes from embryo images, given the critical need for both precision and recall in medical diagnostic processes.

### 3.3 DeepEmbryo results

A performance evaluation of the proposed method is presented in this section based on the metrics presented in Section 3.2. In order to accomplish this, SI-DeepEmbryo and DeepEmbryo algorithms without pre-trained weights were evaluated and compared in the first part of this section. This section of the paper also evaluates the original SI-DeepEmbryo and DeepEmbryo (with TL).

In order to prove the effectiveness of Transfer Learning, the results of SI-DeepEmbryo and DeepEmbryo with no pre-trained architecture has shown in Table 2.

**Table 2:** Results of using architectures without pre-trained weights in DeemEmbryo and SI-DeepEmbryo.

DeepEmbryo Type	AlexNet	ResNet18	ResNet34	Inception V3	DenseNet121
SI-DeepEmbryo	Not learned	Not learned	Not learned	58.33%	56.77%
DeepEmbryo	Not learned	58.33%	Overfit	66.67%	Overfit

As shown in Table 2, SI-DeepEmbryo with three architectures (AlexNet, ResNet18, ResNet34) cannot learn from data which means they classify all samples in one class. SI-DeepEmbryo achieved an accuracy of 58 and 56% with Inception V3 and DenseNet121, respectively. DeepEmbryo cannot classify instances using AlexNet and predict the same class for all instances. DeepEmbryo overfits when it uses ResNet18 and DensNet121. When using ResNet34 and Inception V3, DeepEmbryo can achieve 58 and 67% accuracies, respectively.

When it comes to using Transfer Learning, The SI-DeepEmbryo makes highly accurate classifications of embryo images. It achieves an accuracy of 69.44% by using only a single embryo image. The Accuracy of DeepEmbryo is even better, and it can classify embryos with an accuracy of 75.0%. In this section, we investigate obtained results.

The segmentation step of DeepEmbryo can differentiate between embryo and non-embryo with a Dice Coefficient of 93.21%. This high score shows that the DeepEmbryo can almost always detect and show the exact border of embryos in the IVF images.

After segmentation, the classification step of DeepEmbryo can predict which embryo results in pregnancy with high Accuracy. Table 3 compares the performance of the DeepEmbryo when it uses different well-known CNN architectures. According to these results, the SI-DeepEmbryo does not perform the same when using different pre-trained CNN architectures. In particular, *ResNet18* outperforms other proposed models in *Recall* and *Accuracy. ResNet34* obtained the best *Precision* and obtained second-best in *Recall* and *Accuracy*.

**Table 3:** SI-DeepEmbryo results with different CNN architecture and transfer learning, bold and italic indicate best and second-best, respectively.

	AlexNet	ResNet18	ResNet34	Inception V3	DenseNet121
Recall (%)	55.55	**66.67**	*63.88*	61.11	58.33
Precision (%)	56.92	66.87	**68.51**	61.25	*67.41*
Accuracy (%)	55.55	**66.67**	*63.88*	61.11	58.33

Table 4 shows that DeepEmbryo gained better results in comparison with SI-DeepEmbryo while using any CNN architecture. The best result of DeepEmbryo is when it uses pre-trained Inception V3. In particular, the best results of DeepEmbryo are better than the best results of SI-DeepEmbryo by 8.33% in *Accuracy*, 10.94% in *Precision*, and 8.33% in *Recall*.

**Table 4:** DeepEmbryo Results with Different Well-known CNN and Transfer Learning.

	AlexNet	ResNet18	ResNet34	Inception V3	DenseNet121
Recall (%)	66.66	75.00	69.88	**75.00**	69.44
Precision (%)	76.00	75.71	69.93	**79.45**	72.90
Accuracy (%)	66.66	75.00	69.88	**75.00**	69.44

To consider the potential risk of overfitting associated with our initial 66.6/33.3 training/testing split, we conducted further experiments to evaluate the impact of varying the proportions of training and testing datasets on the model’s performance. We systematically tested additional splits of 70/30 and 80/20 to assess any potential improvements or detriments in model accuracy and generalization capability. The comparative analysis revealed that the differences in performance metrics across these splits were statistically insignificant. This indicates that while our initial split provided a sufficient quantity of data for training without leading to overfitting, altering the proportion of the dataset allocated to training versus testing does not significantly affect the robustness or the predictive accuracy of our models. These findings support the adequacy of our initial data partitioning strategy, ensuring that the model is both effective and efficient in utilizing the available data for training and validation purposes.

### 3.4 Human assessment

Five senior-level embryologists, each with a minimum of 5 years of experience from three different clinics, were involved in our study. They evaluated all embryo images within the test set of the DeepEmbryo system (three images for each embryo), providing critical assessments based on their extensive expertise.

Embryologists used implantation potential (implanted or not implanted) to label the embryo pictures. The accuracies of both SI-DeepEmbryo and DeepEmbryo are better than all embryologists. The embryologists were not in complete agreement on how to classify the test set images, so we used a majority voting procedure. While the majority votes of embryologists reached an accuracy of 48.41%, each of them predicted the pregnancy results of embryos with 54.76, 50.79, 60.32, 60.32, and 39.68%, respectively (Table 5).

**Table 5:** Comparison between expert embryologist and DeepEmbryo and SI-DeepEmbryo.

	Embryologist 1	Embryologist 2	Embryologist 3	Embryologist 4	Embryologist 5	Majority Embryologist	SI-DeepEmbryo	DeepEmbryo
Recall	55.00	59.72	53.89	53.06	46.11	53.06	*66.66*	**75.00**
Precision	54.09	58.93	53.57	52.88	46.43	52.89	*66.87*	**79.45**
Accuracy (%)	54.76	50.79	60.32	60.32	39.68	48.41	*66.67*	**75.00**

## 4 Discussion

Infertility has become a global health concern that affects people all over the world. Even though ART has made significant progress with its extensive worldwide development, the global infertility rate remains high.

The final morphology and morphology during embryo development are essential in detecting the best embryo for transfer. Routinely, sequential cleavage embryo assessment is considered embryo morphology in 19 ± 1, 43 ± 1, and 67 ± 1 hpi (Sakkas and Gardner, 2017). DeepEmbryo uses images belonging to 19, 42, and 66 hpi, which means it fits the current process in IVF labs. No additional work needs for embryologists to use DeepEmbryo in their labs.

Several recent studies have used AI algorithms for grading or predicting the implantation potential of blastocyst-stage (extended embryo culture) embryos (Chen et al., 2019; Khosravi et al., 2019; Bormann et al., 2020; Chavez-Badiola et al., 2020). Extended embryo culture until the blastocyst stage may not be beneficial for all patients. The main drawback of prolonged culture for blastocyst transfer is an increase in the percentage of patients who do not have an embryo for transfer (from 2.9% on day 3 to 6.7% on day 5) (Gardner and Lane, 2017). Hence, many ART clinics use the cleavage embryo transfer strategy yet. An algorithm designed to work based on cleavage-stage embryo images makes it useful for all ART clinics.

In this study, we used a kind of combined algorithm (DeepEmbryo) to schedule time points to detect morphology features and the process of cleavage embryo development. The results show that DeepEmbryo can extract features from three different embryo images and predict pregnancy test results based on those features.

In the absence of objective, standardized criteria, it may be challenging to arrive at an accurate and efficient diagnosis and subsequent prognosis. Presently blastocyst selection is facing a similar problem since most accepted classifications rely on subjective evaluative processes performed by embryologists. A non-morphokinetic classification is also based on characteristics that do not require measurements, perhaps for the sake of simplicity. In this way, they ignore variables or characteristics that cannot be clearly identified by the naked eye but could be indicative of development potential.

Transfer learning can help DeepEmbryo to learn even with limited data. Without transfer learning, DeepEmbryo could not tune millions of parameters with a limited medical dataset. With transfer learning, DeepEmbryo only changes parameters related to a few last layers, which means DeepEmbryo can converge even with a limited dataset.

Image segmentation also helped a lot in the simplicity of input data. While original images had many pixels related to the culture medium, the segmented part only has pixels related to the embryo. Furthermore, embryo location is not fixed in all images. For example, it is at the bottom of the image in one training instance and at the top of the image in another instance. So by eliminating parts of images that are unrelated to the embryo, DeepEmbryo can learn better from data.

Unlike the naked eye, computer-aided image processing tools can detect key image characteristics in a fast, objective, and replicable way. In this study, we compare the performance of our model with 5 embryologists. In the future, this technology will enter the laboratories and be used before selecting the embryo for implantation into the uterus so that embryos with a higher chance of pregnancy can be suggested to the embryologist. There were several limitations to this study. For example, it was not possible to collect data from several centers and data was collected from only one center. Also, due to the nature of the model, it is not possible to interpret the results. The utilization of embryo images in 19 ± 1, 43 ± 1, and 67 ± 1 hpi, which are routinely used for sequential cleavage embryo morphology assessment in IVF labs, was a strength of the current study. Using these images, there is no need to perform additional work or modify standard operating procedures in order to apply DeepEmbryo in embryology labs. Moreover, we comparing the performance of models with five senior-level embryologists in order to validate models.

According to our results, AI-based deep learning tools can be promising for investigating embryo characteristics to predict pregnancy and as an ideal candidate for the embryo selection method in the future. More studies need to be performed using a larger population pool to validate the prediction model and subsequent clinical applications. Multicenter data can help in this regard substantially. It may be helpful to combine the clinical information, including clinical details of the patient, with images of the embryos for a more accurate pregnancy prediction.

## 5 Conclusion

This study has successfully introduced DeepEmbryo, an advanced AI-based algorithm designed to refine the process of embryo selection in IVF treatments. Utilizing a novel approach by analyzing three static images captured at distinct post-insemination intervals, DeepEmbryo employs renowned CNN architectures with the augmentation of transfer learning. This method has demonstrated a substantial improvement in predicting pregnancy outcomes, significantly surpassing traditional morphological assessments and evaluations made by experienced embryologists.

Our results reveal that DeepEmbryo, by integrating multiple time-point images, can achieve an accuracy rate of up to 75.0% in forecasting pregnancy success. This multi-image analysis approach underscores the immense potential of AI to transform embryo selection into a more objective, reliable, and precise process. Importantly, DeepEmbryo’s methodology is compatible with current IVF lab procedures, requiring no additional modifications to existing workflows or equipment, thus facilitating its seamless integration into clinical practice.

The comparative analysis across different CNN architectures and the investigation into the optimal data split for training and testing have yielded valuable insights into leveraging AI in IVF contexts. Furthermore, the inclusion of human assessments in our study accentuates the subjective variability present in conventional embryo evaluation methods and bolsters the argument for adopting a standardized, AI-driven evaluation model.

Despite the promising outcomes, this study’s limitations, such as the singular center data source and the absence of multicenter data, must be acknowledged. Future research should focus on corroborating the efficacy of DeepEmbryo across diverse datasets and examining the potential of merging clinical patient data with embryo imaging for an even more refined prediction of pregnancy outcomes.

DeepEmbryo stands as a pivotal advancement in applying AI to assist reproductive technologies, offering the prospect of enhancing IVF success rates through improved embryo selection. By moving toward a data-driven decision-making paradigm that leverages the comprehensive analysis of multiple embryo images, DeepEmbryo paves the way for supporting couples on their path to parenthood with greater certainty and optimism.

## Data availability statement

The original contributions presented in the study are included in the article/supplementary material, further inquiries can be directed to the corresponding author.

## Ethics statement

The studies involving humans were approved by Research Ethics Committee of Tarbiat Modares University. The studies were conducted in accordance with the local legislation and institutional requirements. The participants provided their written informed consent to participate in this study.

## Author contributions

M-RB: Conceptualization, Formal analysis, Software, Validation, Writing – original draft. MS: Writing – review & editing. BM: Data curation, Writing – review & editing.
